# Plasticity of intrinsic excitability in mature granule cells of the dentate gyrus

**DOI:** 10.1038/srep21615

**Published:** 2016-02-09

**Authors:** Jeffrey Lopez-Rojas, Martin Heine, Michael R. Kreutz

**Affiliations:** 1 Research Group ‘Neuroplasticity’, Leibniz Institute for Neurobiology, Brenneckestrasse 6, D-39118 Magdeburg, Germany; 2 Research Group ‘Molecular Physiology’, Leibniz Institute for Neurobiology, Brenneckestrasse 6, D-39118 Magdeburg, Germany; 3 Leibniz Group ‘Dendritic Organelles and Synaptic Function’, University Medical Center Hamburg-Eppendorf, Center for Molecular Neurobiology, ZMNH, 20251 Hamburg, Germany

## Abstract

The dentate gyrus is the main entry gate for cortical input to the hippocampus and one of the few brain areas where adult neurogenesis occurs. Several studies have shown that it is relatively difficult to induce synaptic plasticity in mature but not in newborn dentate granule cells. In the present work we have systematically addressed how classical protocols to induce synaptic plasticity affect action potential firing and intrinsic excitability in mature granule cells. We found that stimulation paradigms considered to be relevant for learning processes consistently modified the probability to generate action potentials in response to a given synaptic input in mature cells, in some paradigms even without any modification of synaptic strength. Collectively the results suggest that plasticity of intrinsic dendritic excitability has a lower induction-threshold than synaptic plasticity in mature granule cells and that this form of plasticity might be an important mechanism by which mature granule cells contribute to hippocampal function.

The hippocampus integrates and temporally stores information from a variety of subcortical and cortical areas. A major input arises from the entorhinal cortex and the dentate gyrus is the main gate through which this highly processed information enters the hippocampus^1^. The principal cells of the dentate gyrus, the granule cells, are a heterogeneous population because the dentate gyrus is one of the few brain areas where neurogenesis persists throughout adulthood. Granule cells of different maturation stages show distinctive morphological and functional features^2^,^3^ and it has been shown in several studies that it is difficult to induce synaptic plasticity in granule cells following maturation, i.e. stronger induction protocols are needed and the actual amount of potentiation is reduced^4^,^5^,^6^,^7^. Since it is generally believed that Hebbian-type of synaptic plasticity is the major mechanism for temporary information storage and memory formation at hippocampal synapses some authors even stated that mature granule cells can be considered as functionally retired and that plastic properties of the dentate are mainly associated with newborn granule cells^8^. However, mature granule cells presumably represent 90–95% of the entire granule cell population. Thus, the question arises whether they might contribute activity-dependent changes in neuronal plasticity that go beyond classical Hebbian-type of mechanisms.

Already the first description of long-term potentiation (LTP) indicated that synaptic plasticity might not be the sole determinant of functional plasticity of granule cells in the dentate gyrus. In their seminal studies Bliss and collaborators observed that occasionally a lasting increase in neuronal excitability occurred without any sign of synaptic potentiation^9^,^10^. This plasticity of excitability has been largely neglected, especially in the dentate gyrus, despite the fact that action potential generation is the essential output of neuronal activity and that the ability to fire action potentials depends not only on excitatory synaptic drive, but also on inhibitory control and intrinsic membrane properties^11^,^12^. Earlier *in vivo* studies found prominent changes of GABAergic inhibition in the dentate after tetanization and also a GABAergic-independent increase in excitability^13^,^14^,^15^. However, in particular at the single cell level studies of this form of plasticity in granule cells are scarce. We therefore explored this phenomenon in the dentate and investigated whether non-synaptic forms of plasticity are prominent in mature granule cells.

## Results

### Excitability changes are inversely related to synaptic potentiation in the dentate gyrus after theta-burst stimulation

**Figure 1:**
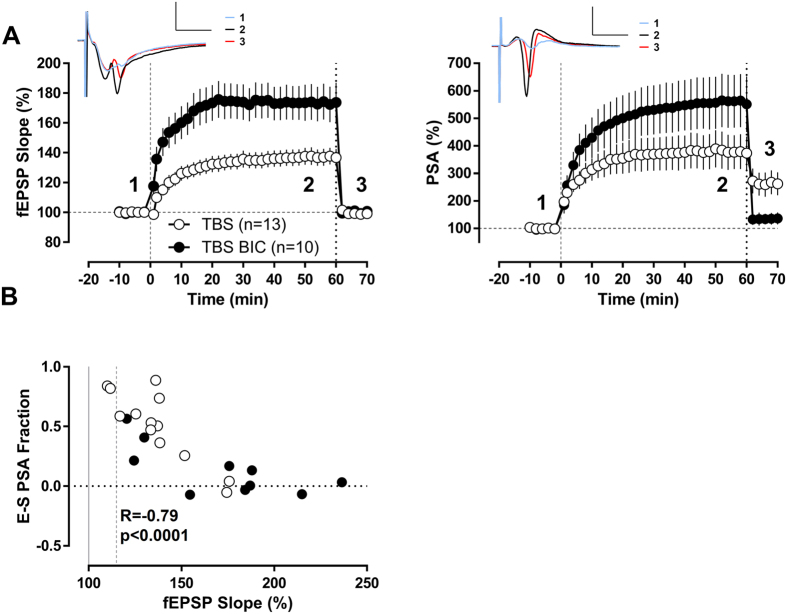
Excitability changes are inversely related to synaptic potentiation in the granule cell population after TBS conditioning protocol. (**A**) Temporal course of population spike amplitude (PSA) and fEPSP-slope. PSA and fEPSP values were normalized for every slice by its pre-conditioning protocol baseline. TBS protocol was applied at time “0” with intact GABAergic inhibition (TBS n = 13) or under bicuculline (TBS BIC n = 10). One hour after TBS protocol, the test stimulation intensity was reduced to match the baseline fEPSP-slope values. The remaining PSA potentiation displays the excitability changes non-explained by the synaptic potentiation. Insets show traces corresponding to the TBS group at the indicated time points: 1- at baseline, 2−1 h after TBS and 3- after reducing stimulation intensity to match baseline fEPSP-slope. Scale bars: 2 mV/5 ms. (**B**) Scatter plot of the fraction of the total PSA potentiation that is not explained by the synaptic LTP (the remaining population spike potentiation after reducing stimulation intensity to match baseline fEPSP-slope was normalized in every slice to the spike potentiation averaged in the last 4 min previous to the stimulation intensity reduction) and the fEPSP-slope potentiation at 1 hour after TBS for every experiment. There was a significant negative correlation between both variables when GABAergic inhibition was intact (R = −0.86, p < 0.0001, Pearson) as well as in the presence of bicuculline (R = −0.71, p < 0.05, Pearson). When both conditions were analyzed together as a whole group the correlation was still highly significant (R = −0.78, p < 0.0001, Pearson).

Theta-burst stimulation (TBS) is designed to mimic the firing patterns of hippocampal neurons recorded during exploratory behaviour in intact awake animals and is an effective protocol for inducing robust and persistent LTP. We therefore first studied the effects of TBS^7^ on the field excitatory postsynaptic potential (fEPSP) in the dentate gyrus and found a modest potentiation 1 hour after TBS (136.8 ± 5.8%) (Fig. 1). Changes in the slope of the fEPSP are considered to reflect changes in synaptic strength and thereby excitatory drive. To next address which contribution this change in synaptic strength has on action potential generation we dissected alterations in the population spike amplitude induced by potentiation of excitatory synapses from excitability changes independent of excitatory synaptic drive. To this end, we decreased the test stimulation intensity 1 hour after TBS in order to obtain fEPSP-slope values similar in magnitude to those recorded at baseline. This allowed us to compare the response of neurons in the dentate gyrus to the same excitatory drive before and after TBS. Most important when we analysed the data we realized that a significant part of the increase in population spike amplitude was not explained by concomitant changes in synaptic potentiation (Fig. 1A). The contribution of components to the increase in population spike amplitude that cannot be explained by alterations in excitatory drive amounted to 50% (Fig. 1A).

Earlier work has suggested that reducing the stimulation intensity not only reduces the excitatory drive to principal neurons, but also the feedforward inhibitory drive^16^. Thus, a change in the excitation-inhibition balance of the neuronal network in the dentate could underlie the observed phenomenon. In this case it is expected that a direct relation exists between fEPSP-slope potentiation and increases in population spike amplitude that are not related to changes in excitatory input. However, in stark contrast to this assumption the analysis of this relation showed a highly significant negative correlation (Fig. 1B, R = −0.86, p < 0.001, Pearson). Similar results were obtained when analysed the E-S relation of single experiments derived from input-output curves (Supplementary Fig. S1). This finding makes it unlikely that exclusively excitatory and inhibitory input in the network are altered and provided first evidence that non-synaptic mechanism might be involved.

Therefore, we decided to study next whether TBS induced changes in excitability are indeed independent of inhibitory input and performed experiments in the presence of bicuculline. As shown previously^17^,^18^, blocking GABA_A_ receptors resulted in larger synaptic potentiation (173.7 ± 13.3% 60 min after TBS n = 10) than under control conditions (Fig. 1A, note also the higher number of single experiments with high fEPSP-slope potentiation in the bicuculline condition in Fig. 1B). Interestingly, the negative correlation between the fEPSP-slope potentiation and the increase in population spike amplitude that is not related to changes in excitatory input was still present (R = −0.71, p = 0.0208, Pearson) when we reduced inhibition (Fig. 1B). Accordingly, the remaining increase of population spike amplitude after reducing the test stimulation intensity to match fEPSP-baseline values before TBS was significantly smaller (ratio of the remaining population spike potentiation to the spike potentiation averaged in the last 4 min previous to the stimulation intensity reduction: 0.14 ± 0.07) than under conditions with intact GABAergic inhibition (Fig. 1A,B). These results suggest that intrinsic properties of granule cells might have been modified by the TBS paradigm.

### LTP in mature granule cells is only induced by theta-burst stimulation and high-frequency stimulation/pairing protocols

The negative relation between fEPSP-potentiation and changes in population spike amplitude that are not related to changes in excitatory input was present in conditions of intact and reduced inhibition. In single experiments we found significant E-S potentiation in presence or absence of bicuculline when the fEPSP-slope potentiation was moderate. However in both groups higher fEPSP-slope potentiation levels corresponded to no increase in excitability. We therefore reasoned that these results might point to changes in intrinsic excitability in mature granule cells that significantly contribute to field responses. The negative correlation also suggests that mild stimulation protocols would be more efficient to increase granule cells intrinsic excitability. To test these hypotheses we recorded in subsequent experiments from single mature granule cells in the presence of the GABA_A_ antagonist bicuculline, in order to study their intrinsic plasticity. We obtained perforated patches from neurons of the upper blade of the dentate gyrus and considered, in accord to the literature, neurons in the outer part of the granule cell layer with an input resistance below 300 MΩ to be mature granule cells^5^,^6^,^7^. The input resistance of neurons recorded in this manner indeed averaged 174.3 ± 3.1 MΩ (minimum-maximum: 112.5 MΩ−265.1 MΩ). To assess whether non-synaptic forms of plasticity can be induced in mature granule cells we then studied the effects of three extensively used LTP-inducing protocols, TBS (similar to the one used in the field recordings), a high-frequency stimulation/pairing protocol (HFS-P) and spike-timing dependent plasticity (STDP) on synaptic strength and intrinsic excitability.

**Figure 2:**
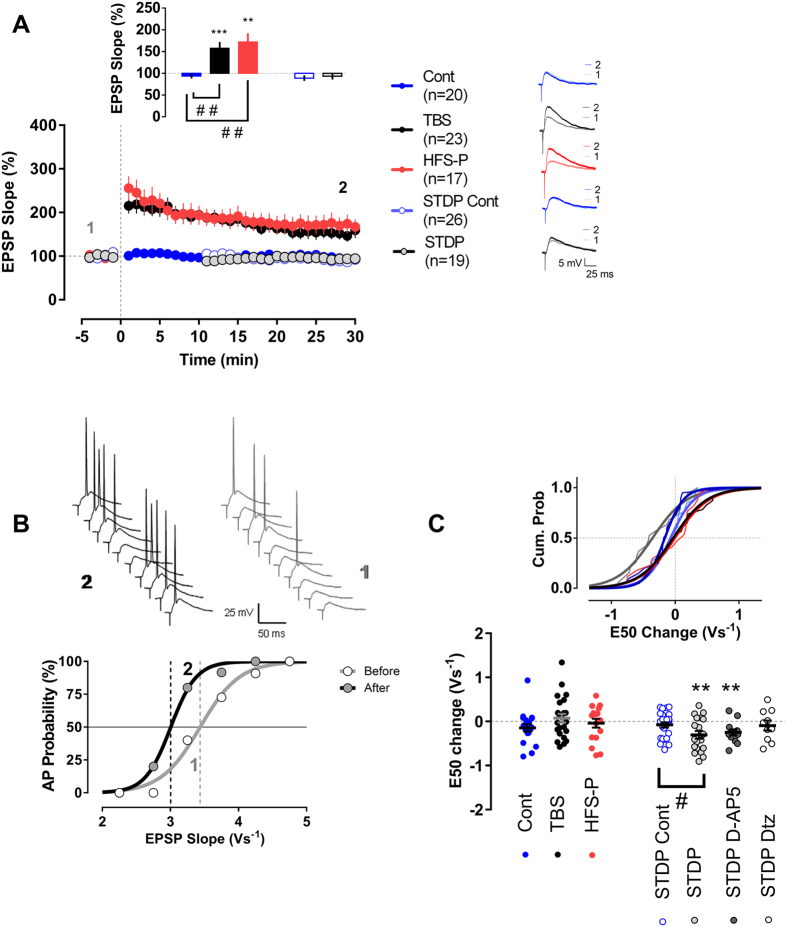
Dissociation between synaptic and excitability plasticity in mature granule cells. (**A**) Synaptic changes induced by the three conditioning protocols (TBS n = 23, HFS-P n = 17 and STDP n = 19) and their respective control groups (Cont n = 20 and STDP-Cont n = 26). EPSP-slope values were normalized for every cell by its pre-conditioning protocol baseline. The insets show the EPSP-slope potentiation in the last 4 min of recordings and representative EPSP traces for the groups. TBS and HFS-P groups showed a significant synaptic potentiation, whereas in the other experimental groups the EPSP-slope remained around baseline values. (**B**) Example of an E-S curve, plotting of the firing probability versus EPSP-slope, for a cell in the STDP group. (**C**) E-S shift. Two E-S curves were generated for every cell before and after (30–45 min) the conditioning protocol. A significant E50 shift in the E-S relation was only present in the STDP group and not in TBS or HFS-P group. This STDP E50 change did not reached statistical significance in the presence of the L-type channel blocker diltiazem (Dtz, n = 10), but was highly significant in the presence of the NMDA receptor antagonist D-AP5 (n = 13). The inset displays the cumulative probability curves for the E50 shifts in Cont, TBS, HFS-P and STDP groups. All recordings were made in the presence of bicuculline. **,***p < 0.01 and p < 0.001, paired t-test. ^#^p < 0.05, t-test. ^##^p < 0.01, Bonferroni post-hoc test after one-Way ANOVA.

From the three protocols tested, only TBS and HFS-P produce LTP in mature granule cells. The EPSP-slope potentiation reached 157.6 ± 14.5% and 171.9 6 ± 20.1% in the last 4 min of recordings for the TBS and HFS-P groups, respectively (Fig. 2A). These results are in agreement with previous studies showing that TBS^7^ and HFS-P^6^,^7^, which are considered to be strong LTP-inducing protocols, indeed induce synaptic potentiation in mature granule cells. In contrast to TBS and HFS-P, STDP has been scarcely studied in the dentate gyrus, especially at the single cell level^19^,^20^,^21^. We adapted a previously published STDP protocol^20^ and found that this STDP protocol failed to induce any significant change in the EPSP-slope of patched mature granule cells. The EPSP-value in the last 4 min of recordings was similar to corresponding controls (Fig. 2A). We also included two further control groups for the STDP group. The first group only received synaptic stimulation (0.166 Hz/STDP-EPSP-Cont n = 13) during the period of STDP-induction whereas the second group only received the postsynaptic current injection (0.166 Hz/STDP-2AP-Cont n = 13) required to elicit two action potentials. Neither synaptic stimulation nor postsynaptic action potential firing alone, provoked any statistically significant changes in the time course of the EPSP-slope or intrinsic excitability (Supplementary Fig. S2). Since we found no differences we merged the latter two control groups for further comparison.

### Synaptic potentiation and changes in intrinsic excitability are not strictly correlated in mature granule cells

However, further analyses then revealed that in contrast to the two strong conditioning protocols that are capable to induce changes in synaptic strength, only the STDP protocol induced a significant modulation of the E-S curve, which relates EPSP-slopes to the associated action potential firing probability (see Fig. 2B for a typical recording from a single cell). A moderate, but highly significant shift to the left of −0.30 ± 0.09 Vs^−1^ in the E50 value (the value of the EPSP-slope that elicits an action potential with 50% of probability) was present in this group (p = 0.003, t = 3.435 df = 18, paired t-test). The shift in E50 that we observed following the STDP protocol was significantly different from the corresponding control group (p = 0.0283, t = 2.269 df = 43, t-test) and E50 was not altered following TBS and HFS-P (Fig. 2C), indicating that the STDP protocol elicited changes in intrinsic plasticity of mature granule cells.

We next asked what might be the induction mechanism for the type of plasticity under investigation in the current study and whether a Ca^2+^ -dependent mechanism of neuronal activation is involved. Interestingly, we found that the E50 shift after the STDP protocol seems to be mainly depend on L-type calcium channels and not on NMDA receptors as the E50 change was absent in the presence of 30 μM of the L-type calcium channel blocker diltiazem, but was not blocked by application of 50 μM of the NMDA receptor antagonist D-AP5 (Fig. 2C).

**Figure 3:**
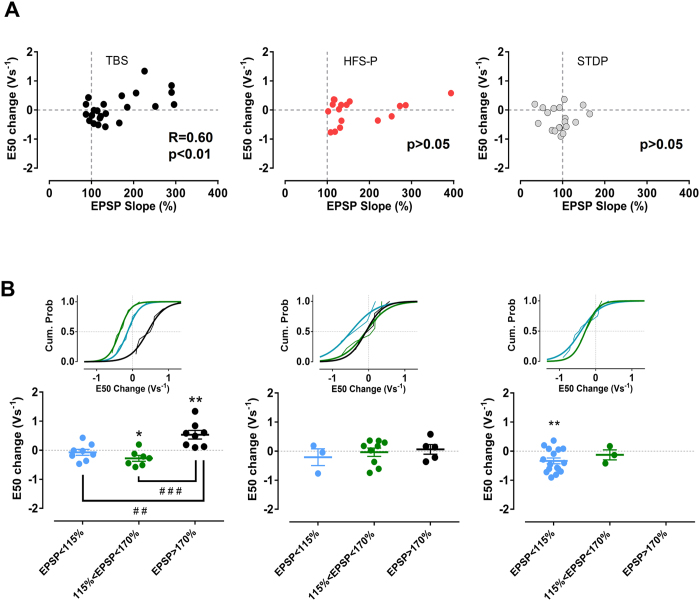
The E50 shift and EPSP-slope potentiation in the TBS group are closely related in mature granule cells. (**A**) Scatter plot of E50 shift and EPSP-slope potentiation. A statistically significant correlation was found in the TBS group. (**B**) When TBS, HFS-P and STDP groups were subdivided according to the EPSP-slope potentiation in the last 4 min of recordings (considering the following criteria: high potentiation EPSP-slope > 170%, moderate potentiation 115% < EPSP-slope < 170% and no potentiation EPSP < 115%), there was a differential E50 shift among the subgroups of TBS. Both the moderate and high EPSP potentiation subgroups displayed a significant E50 shift, but in opposite directions. All recordings were made in the presence of bicuculline. *,**p < 0.05 and p < 0.01, paired t-test. ^##, ###^p < 0.01 and p < 0.001, Bonferroni post-hoc test after one-Way ANOVA.

Even though no change in the E-S relation was observed following TBS, a closer inspection of the data revealed a highly significant correlation between the EPSP-slope potentiation and the E50 shift (R = 0.60, p = 0.0026, Pearson), which was not present following HFS-P or STDP protocol (p > 0.05, Pearson) (Fig. 3A). This correlation was reminiscent of the correlation that we found in the field recording experiments using a comparable TBS stimulation protocol (Fig. 1B). It has been shown previously that TBS modulates excitability of CA1 pyramidal cells according to the level of EPSP induced-potentiation^22^. Interestingly, we observed that, when we assigned neurons after application of TBS to three subgroups according to the EPSP-slope potentiation in the last 4 min of the recording period (considering the following criteria: high potentiation EPSP-slope > 170%; moderate potentiation 115% < EPSP-slope < 170% and no potentiation EPSP < 115%), the subgroup corresponding to the highest potentiation showed a significant E50 shift to the right of 0.53 ± 0.15 Vs^−1^ (p = 0.0089, t = 3.589 df = 7, paired t-test). In contrast, the subgroup showing moderate potentiation exhibited an increase in excitability (E50 shift −0.28 ± 0.10 Vs^−1^; p = 0.0320, t = 2.780 df = 6, paired t-test), whereas no E50 shift was evident in the group showing no EPSP-potentiation (E50 shift −0.07 ± 0.10 Vs^−1^; p > 0.05, paired t-test) (Fig. 3B). Taken together, this analysis further supports the view that changes in the probability to fire action potentials are not directly coupled to EPSP-slope potentiation and thus are regulated at least in part independent of changes in synaptic strength.

### Dendritic excitability is malleable in mature granule cells

**Figure 4:**
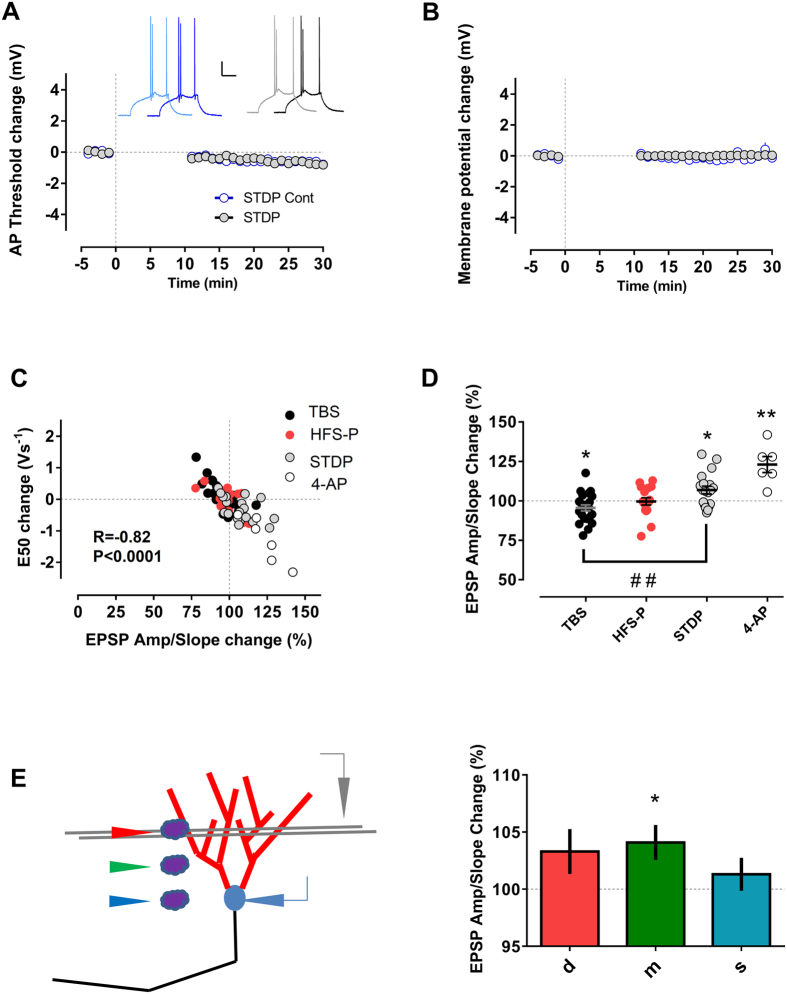
The E50 shift was strongly correlated with the EPSP-amplitude/slope change in all three (TBS, HFS-P and STDP) groups. There were no significant changes in action potential threshold, (**A**) or membrane potential, (**B**) between STDP and STDP control group that could account for the differences in intrinsic plasticity among these groups. In (**A**), the insets show traces of action potentials in the STDP control (blue) and the STDP group (black) before (light color) and around 25 min after (dark color) time “0”. Scale bars: 20 mV/100 ms. (**C**) In every group the correlation between E50 shift and EPSP-amplitude/slope change was statistically significant: TBS R = −0.77, p < 0.0001; HFS-P R = −0.70, p < 0.01 and STDP R = −0.53, p < 0.05, Pearson). In another group of cells (n = 6), blockade of potassium A-type currents with bath application of 1 mM 4-aminopyridine for 30 min significantly changed the EPSP-amplitude/slope relation and this change was highly correlated with the excitability modification (R = −0.93, p < 0.01, Pearson). The correlation was also highly significant when analyzing all groups together (R = −0.82, p < 0.0001, Pearson). (**D**) TBS and STDP groups showed a significant change in the EPSP-amplitude/slope relation, both groups also differed from each other. (**E**) 4-aminopyridine was locally puffed at different positions: close to the stimulated dendritic region (d), at a dendritic region in between the stimulation site and the soma (m), and at the soma (s) of a mature granule cell. The amplitude-slope relation of the EPSP was measured as a readout of dendritic excitability. Only the puff to the dendrites in between the stimulation site and the soma resulted in a significantly enhanced EPSP amplitude-slope relation (n = 28). All recordings were made in the presence of bicuculline. *,**p < 0.05 and p < 0.01, paired t-test. ^##^p < 0.01, Bonferroni post-hoc test after one-Way ANOVA.

In the previous experiments we could not find any significant changes in the threshold for action potential generation or membrane potential (Fig. 4A,B) that could account for the observed excitability changes. We therefore analyzed next the possibility that modified excitability resulted from changes in dendritic processing of synaptic input. One measure of dendritic excitability is the EPSP-amplitude/slope ratio and altering the EPSP-amplitude/slope ratio will have impact on the probability to reach the action potential threshold for a given synaptic input. In the first milliseconds the EPSP-slope mainly reflects the fast component of AMPA-receptor synaptic transmission, whereas the EPSP-amplitude is also strongly influenced by voltage-gated dendritic ion channels that are recruited later on, as for instance the A-type potassium current^23^,^24^. Accordingly, it has been shown that modulation of the A-type current has profound implications for neuronal excitability in CA1 pyramidal neurons^23^,^25^,^26^. A-type potassium channels are also expressed in dendrites of granule cells^27^ and it is known that they can be modulated by activity in the dentate gyrus^28^,^29^. To test the hypothesis that A-type conductance contributes to the observed intrinsic plasticity phenomena in mature granule cells we first evaluated the consequences of a pharmacological blockade of A-type channels on EPSP-amplitude/slope ratio and intrinsic excitability on mature granule cells. Bath application of 1 mM of the A-type potassium channel blocker 4-aminopyridine for 30 min resulted indeed in an increment of the EPSP-amplitude/slope relation to 123.1 ± 5.1% and a significant shift in the E50 value of −1.29 ± 0.30 Vs^−1^. Interestingly, both measures were highly correlated (Fig. 4C,D, R = −0.93, p = 0.0064, Pearson). In another series of experiments, puffing 4-aminopyridine 10 mM locally to dendrites of mature granule cells (around 50 μm from soma, a location between the stimulation site and the soma) significantly modified as well the EPSP-amplitude/slope relation to 104.2 ± 1.5% (n = 28 cells, p = 0.0321, t = 2.26 df = 27, paired t-test), whereas puffing to the soma did not affect this relation (101.5 ± 1.4%, n = 28 cells, p = 0.6661, t = 0.4362 df = 27, paired t-test/ Fig. 4E). Thus, changes in the EPSP-amplitude/slope relation have probably a prominent origin in proximal dendrites.

We then asked if the EPSP-amplitude/slope relation was also modified by the other conditioning protocols (TBS, HFS-P and STDP, see above) and how such changes are related to neuronal excitability. Changes in E-S were indeed highly correlated with EPSP-amplitude/slope modulation in all groups (Fig. 4C, R = −0.72, p < 0.0001, Pearson). In most recordings, a left shift in E50 was accompanied with an increased EPSP-amplitude/slope ratio and a right E50 shift with a reduction of the EPSP-amplitude/slope. However, only the TBS and STDP protocols induced statistically significant changes in the EPSP-amplitude/slope relation. The EPSP-amplitude/slope relation was enhanced in the STDP (106.9 ± 2.4%; p = 0.0094, t = 2.909 df = 18, paired t-test) and reduced in the TBS group (Fig. 4D, 95.6 ± 1.9%; p = 0.0173, t = 2.574 df = 22, paired t-test).

**Figure 5:**
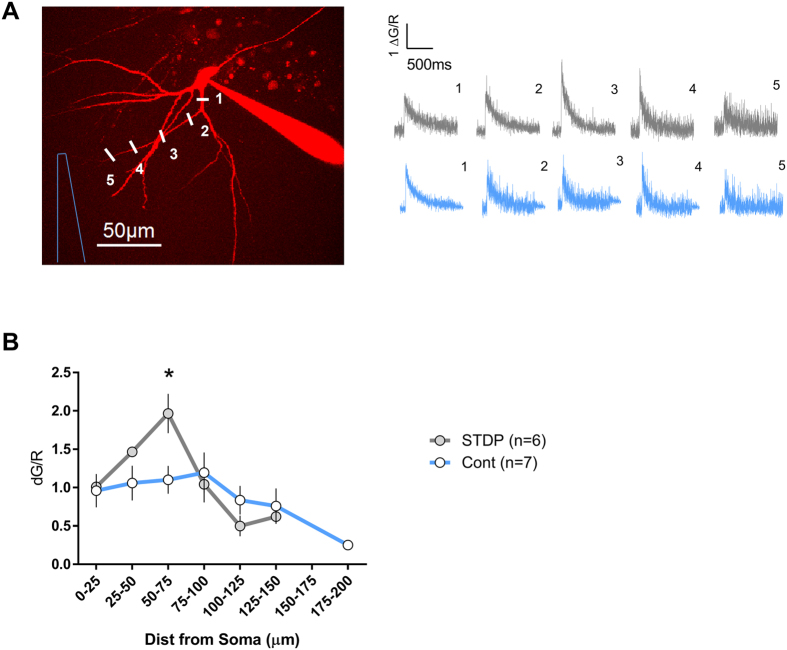
Local boosting of Ca^2+^ influx evoked by 1 backpropagating action potential in dendrites of mature granule cells after STDP conditioning protocol. (**A**) Two photon fluorescence image of a mature granule cell. The inset shows representative Ca^2+^ transients (corresponding to the points marked in the fluorescence image in the case of the gray traces, and to similar distances for the blue traces that are corresponding to another cell in control condition) around 15–20 min after attaining the whole cell configuration. A field stimulation electrode was placed 100 μm from the soma and the closest dendrite was chosen for the measurements. (**B**) Profiles of backpropagating action potential-induced Ca^2+ ^influx along dendrites of cells that received the STDP protocol (n = 6) and another group of cells with no STDP (n = 7). All recordings were made in the presence of bicuculline. *p < 0.05 Bonferroni post-hoc test after two-Way ANOVA.

Taken together changes in EPSP-amplitude/slope ratio and their close relation to alterations in E50, indicate that modifications of A-type channels mainly in proximal dendrites might underlie the observed alterations in intrinsic excitability. It has been shown that the modulation of dendritic ion channel conductances, like A-type, does not only reshape the EPSP waveform, but also alters the backpropagating action potential amplitude^25^,^30^. Therefore, we next tested whether changes in the backpropagating action potential-induced Ca^2+^ transients, indirectly reflecting the amplitude of the backpropagating action potential, occur in dendrites of mature granule cells after the STDP protocol. A field stimulation electrode was positioned in the molecular layer, approximately 100 μm away from the soma, close to the potential dendritic region of a targeted granule cell. We then patched the granule cell in the whole cell configuration (pipette solution included the volume marker Alexa 594 and the Ca^2+^ indicator Fluo 5F) and immediately applied the STDP protocol. Five minutes following the induction of STDP we chose the dendrite closest to the stimulation electrode and measured the Ca^2+^ transients along the chosen dendrite in response to one backpropagating action potential. As shown in Fig. 5, the STDP group exhibited a different spatial profile of Ca^2+^ transients as compared to the control group. Notably, in dendritic segments 50–75 μm away from the soma, roughly the region where puff application of 4-aminopyridine modifies the EPSP-amplitude/slope relation, an approximately 2-fold increase of a single backpropagating action potential induced-Ca^2+^ influx was evoked after the STDP protocol as compared to controls. This increase could reflect downregulation of the A-type currents in this dendritic segment and together with the changes in EPSP-amplitude/slope ratio described above, these data are also in support of the notion that modulation of the A-type potassium might alter intrinsic excitability in dendrites of mature granule cells.

Based on this evidence we tested next whether pharmacological blockade of A-type potassium channels occludes the observed intrinsic plasticity of mature granule cells (Supplementary Fig. S3). Bath application of 4-aminopyridine completely prevented the increase in excitability, as well as the correlation between the fEPSP potentiation and the population spike increase not explained by concomitant changes in synaptic potentiation, after the TBS protocol in field recordings. Intrinsic changes were also occluded by 4-aminopyridine after STDP protocol in single mature granule cells. No significant change in the E-S curve, relating EPSP-slopes to the associated action potential firing probability, occurred in the group where 4-aminopyridine was bath applied (E50 change: 0.076 ± 0.16 Vs^−1^, n = 9). Accordingly, the EPSP-amplitude/slope relation was also not significantly modified in this group (EPSP-amplitude/slope relation change: 100.9 ± 5.1%, n = 9) (Supplementary Fig. S3).

### Supralinear Ca^2+^ summation in spines and dendrites of mature granule cells during STDP pairing

Pyramidal CA1 neurons can detect the temporal coincidence of input (EPSP) and output signals (backpropagating action potential) based on changes in Ca^2+^ dynamics^31^,^32^. However, it is not known whether mature granule cells of the dentate gyrus exhibit similar properties. Therefore, using two-photon microscopy we measured how Ca^2+^ -summation is affected in spines and dendrites of mature granule cells during STDP protocol pairings. We measured the Ca^2+^ transients in response to a sub-threshold EPSP evoked by a field stimulation electrode positioned close to the target spine, a current injection evoking a duplet of action potentials or a pairing consisting on a combination of both EPSP and action potentials, similar to the STDP pairing described above. The ratio of the Ca^2+^ transients observed with the pairings and the linearly summed values of the transients for the single stimuli (EPSP and action potentials) was used as a measure of supralinearity. Remarkably, the pairings resulted in a significant nonlinear Ca^2+^ summation in spines (1.23 ± 0.06; p = 0.0008, t = 3.939 df = 20, one sample t-test vs. 1) as well as in their parent dendrites (1.15 ± 0.05; p = 0.0076, t = 3.029 df = 17, one sample t-test vs. 1) (Supplementary Fig. S4).

## Discussion

**Figure 6:**
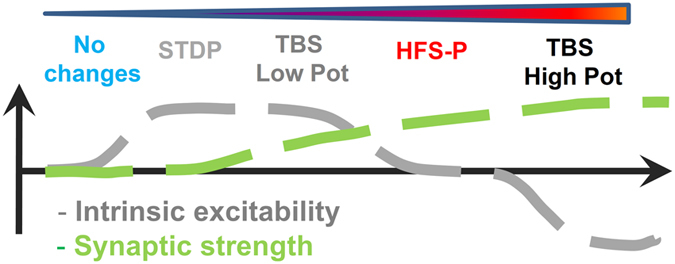
Proposed model for the relation among synaptic and intrinsic plasticity in mature granule cells. We propose that intrinsic plasticity might be the predominant form of plasticity triggered by mild conditioning protocols in mature granule cells. In the case of stronger induction protocols, changes of excitability in opposite directions can be elicited depending on the amount of synaptic potentiation achieved.

In this paper we describe a surprising malleability in the capacity of dentate gyrus neurons to fire action potentials in response to a given excitatory drive after TBS. We observed that this plasticity of excitability was inversely correlated to the degree of synaptic potentiation and that it can account for an astonishingly large part of alterations in the population spike amplitude following moderate fEPSP potentiation. We therefore asked whether these changes in excitability could reflect a novel, scarcely explored, form of plasticity of mature granule cells, the major cell population in the dentate gyrus. In subsequent experiments we indeed found a bidirectional form of non-synaptic plasticity in these neurons. Mature granule cells exhibit increased intrinsic excitability in response to mild conditioning protocols, while stronger EPSP-potentiation has the opposite effect. Strikingly, a protocol to induce STDP failed to elicit changes in synaptic strength during the recording period but consistently increased excitability, indicated by a left shift in the E-S relation. Based on these results, we propose a model (Fig. 6) in which relatively mild conditioning protocols, such as STDP, have the potential to modify mature granule cells excitability without altering synaptic strength. Stronger protocols, like TBS, need to reach a certain threshold to elicit both, changes in synaptic strength and intrinsic excitability. Once this threshold is reached, the outcome of the excitability change depends on the level of EPSP-potentiation: at moderate EPSP-potentiation levels intrinsic excitability is increased whereas at higher EPSP-potentiation the likelihood to fire action potentials is decreased. HFS-P failed to induce any change in intrinsic excitability despite clear EPSP potentiation. Of note these results contradict Lin *et al*. (2006), who found synaptic potentiation utilizing STDP protocols, but several differences can explain the discrepant results, like the age of the animals, the stimulation of different synaptic inputs (lateral perforant path vs. medial perforant path) as well as the type of recordings (field recordings vs. single cell recordings from mature granule cells). However our results are in agreement with Yang and Dani (2014) who did not find any synaptic potentiation employing a STDP (Δt = + 10 ms) protocol in patched granule cells.

In summary, we propose that non-synaptic plasticity exhibits a lower induction threshold than synaptic plasticity in mature granule cells and that intrinsic plasticity is the predominant mechanism triggered by a mild conditioning protocol in these cells. What could be underlying mechanisms for such changes? We did not observe any significant alteration in the threshold for action potential firing or the membrane potential that could explain the observed changes in intrinsic excitability. A contribution of altered dendritic conductances that allow a differential translation of the synaptic input into depolarization is therefore likely. Indeed, we found a highly significant correlation between the E-S shift and the EPSP-amplitude/slope ratio in all groups and a slight increase of this EPSP-amplitude/slope ratio in the STDP group. A similar change in EPSP-amplitude/slope relation was reported for STDP in CA1^24^. We also found that STDP induced a local boost of Ca^2+^ influx evoked by a backpropagating action potential in dendritic segments close to the stimulation electrode. This local boost is reminiscent of the activity-dependent redistribution of Kv4.2 seen in the dentate gyrus molecular layer of rats that underwent pilocarpine treatment^28^. Together the changes in the EPSP waveform and the local boost of Ca^2+^ transients evoked by a backpropagating action potential are consistent with the idea that downregulation of A-type potassium currents could be a potential mechanism of the observed excitability changes. In support of this notion, we saw that pharmacological blockade of A-type channels produced profound changes in EPSP waveform and excitability of mature granule cells. Moreover, the STDP pairings used in the STDP protocol were associated with a supralinear summation of Ca^2+^ in spines and parent dendrites of mature granule cells. This influx of Ca^2+^ could possibly be important for a subsequent post-translational modification of the A-type channels, which are modulated by PKA, PKC, MAPK and CAMKIIα^33^,^34^,^35^,^36^. Pharmacological experiments showed that the major Ca^2+^-dependent influx pathways are rather L-type Ca^2+^ channels than NMDAR further supporting the observation that high-frequency stimulation of synapses is not a pre-requisite to induce this type of plasticity. Although we have focused on mature granule cells intrinsic plasticity, changes in GABAergic inhibition in the dentate gyrus might also have an important role in dentate functional plasticity as already suggested by earlier^13^,^14^,^15^ and more recent^37^,^38^ work.

A major shortcoming of studying neuronal plasticity *in vitro* is the questionable physiological relevance of the conditioning protocols used to simulate a learning event. HFS-P protocols have been criticized due to the lack of experimental evidence that such long firing periods actually occur *in vivo*^39^,^40^. The same criticism applies at least in part to pairing protocols, where cells are depolarized to positive potentials for long time periods. TBS protocols are presumably physiologically more relevant, as theta oscillations have been related to learning *in vivo*^41^,^42^. STDP has also raised a lot of attention due to the very mild conditions required for its induction and its potential physiological relevance^43^,^44^. Of importance for the present work is also a recent study by Pernia-Andrade and Jonas (2014) that showed, with whole-cell recordings from mature granule cells of awake rats exploring a new environment, that mature cells *in vivo* fired action potentials preferentially in bursts and those bursts occurred on average every 5 s, while exposed to massive functional glutamatergic input from the entorhinal cortex^45^. This is reminiscent of the STDP protocol employed in the current study that includes action potential duplets with pairings every 6 s. We consider it therefore likely that mature granule cells *in vivo* are subject to activation patterns similar to the STDP protocol used in this study and that this activation will not alter synaptic strength in mature granule cells but intrinsic dendritic excitability. Mature granule cells show very strong basal levels of dendritic voltage attenuation, making the contribution of individual synapses to mature granule cells action potential firing quite low^46^. Therefore changing the ‘EPSP-attenuation’ levels by modifying dendritic excitability can plausibly more readily impact on the output of these cells than induction of synaptic potentiation. Thus, we propose that non-synaptic forms of plasticity are probably very important mechanisms through which mature granule cells contribute to hippocampal function.

## Methods

All experimental procedures were carried out in accordance with the EU Council Directive 2010/63/EU and were approved by the local Committee for Ethics and Animal Research (Landesverwaltungsamt Sachsen-Anhalt, Germany).

### Field recordings

#### Hippocampal slices

Field recordings were done in transversal hippocampal slices from 10 weeks old male Wistar rats. The right hippocampus was isolated in ice-cold artificial cerebrospinal fluid (ACSF) solution. Hippocampal slices (400 μm thickness) were cut with a chopper and placed in an interface chamber at 32 °C. The ACSF solution was the same as in the single cell experiments and contained in mM: 124 NaCl, 4.9 KCl, 2 MgSO_4_, 2 CaCl_2_, 1.2 KH_2_PO_4_, 25.6 NaHCO_3_ and 20 glucose, equilibrated with 95% O_2_/5% CO_2_. Slices were incubated for at least 3 hours before the start of the recordings that were performed in the same incubation interface chamber at 32 °C^47^.

#### Electrophysiology

The population spike and the field- excitatory postsynaptic potential were measured with two monopolar lacquer-coated, stainless steel electrodes positioned at the granule cell layer and middle of molecular layer, respectively. One stimulation electrode placed in the middle of the molecular layer was used to stimulate the medial perforant path and a second one placed in the hilus to elicit antidromic spikes by stimulation of the mossy fibers. Biphasic constant current pulses (0.1 ms per half-wave duration) to the perforant path at 0.033 Hz evoking 25% of maximal orthodromic population spike amplitude were used for test recordings.

#### LTP-induction protocol

The LTP-induction protocol was similar to theta-burst stimulation (TBS) used in the single cell experiments and consisted of 4 episodes repeated at 0.1 Hz, each episode including brief presynaptic bursts −10 pulses at 100 Hz− repeated 10 times at 5 Hz. Each presynaptic stimulus (0.2 ms per half-wave duration) was paired with an antidromic stimulus (0.2 ms per half-wave duration; delay of 5 ms between ortho- and antidromic stimulation).

### Single cell recordings

#### Hippocampal slices

Transverse 400 μm slices from the right hippocampus of adult male Wistar rats (10 weeks old) were cut with a vibratome (Leica VT1000S) in ice-cold ACSF solution. Slices were incubated at 34 °C for 25 min and subsequently held at room temperature. The same extracellular solution was used for preparation, incubation and holding of the slices.

#### Electrophysiology

Somatic perforated-patch recordings were made from mature granule cells in the outer part of the granule cell layer of the dentate gyrus upper blade, with an input resistance below 300 MΩ. Patch pipettes (6–12 MΩ) were pulled from thick-walled borosilicate glass tubing. Gramicidin (80 μg/ml final concentration) was daily prepared as stock solution in DMSO and added to the pipette solution containing in mM: 130 potassium gluconate, 20 HEPES, 1 CaCl_2_, 2 MgCl_2_, and 0.1 EGTA. The pH was adjusted to 7.3 and the osmolarity to 290 mOsm. The pipette tip was filled with gramicidin-free solution.

In the recording chamber, slices were initially perfused with the same extracellular solution used in preparation, incubation and holding. 20 μM bicuculline was added approximately 1 hour before the start of the experiment, around 0.5–1 hour after patching the cell, to block GABA_A_ receptors and thereby study mature cell intrinsic properties. Typically, a cell was patched and in the cell-attached configuration small depolarizing pulses were applied (in voltage-clamp) in order to follow the perforation process. After 1.5–2 hours a stable access resistance developed (access resistance change 3.6 ± 1.3% mean ± standard error of the mean). At this point we switched to current-clamp mode. To stimulate the medial perforant path a patch pipette filled with extracellular solution was placed aimed to the central part of the molecular layer close to the dendrites of the recorded cell. Recordings were performed at 25 °C.

At the start of the experiment an E-S (EPSP slope vs. Spike probability) curve was generated by varying the strength of the synaptic stimulation in order to get a representation of the whole range: from an EPSP evoking a spike with 100% probability to sub-threshold EPSPs evoking no action potential. Around 60–70 stimuli were applied at 0.1 Hz. EPSP-slopes were measured during the first 2 ms and sorted in 0.5 Vs^−1^ bins. The firing probability was calculated for each bin and the E50 value was estimated by fitting the curve to a sigmoid function. After this, a stimulation strength giving an EPSP of around 7 mV was chosen for the test recordings, at 0.066 Hz. This synaptic stimulus was followed after 250 ms by a current injection in order to assess possible changes in action potential features. A baseline was recorded for 4 min and then an LTP protocol was applied followed by 30 min (or 20 min in the ‘spike-timing-dependent plasticity’ (STDP) groups, because the STDP protocols lasted for 10 min) of further test recordings. At the end, a new E-S curve was generated in a similar fashion as the initial curve. The sub-threshold EPSPs in the E-S curves were used in the calculation of the EPSP-amplitude/slope ratios. The slope of the EPSP-amplitude vs. EPSP-slope relation was calculated and the ratio Slope-After/Slope-Before is reported as the EPSP-amplitude/slope change. Recordings were performed with an EPC9 patch-clamp amplifier. Data were acquired and stored using Patchmaster and analyzed with Fitmaster and GraphPad Prism.

#### LTP-induction protocols

Three different LTP protocols were employed. The theta-burst protocol (TBS, similar to the one used in the field recording experiments) comprised 4 episodes repeated at 0.1 Hz, each episode consisting of brief presynaptic bursts −10 pulses at 100 Hz- repeated 10 times at 5 Hz. Each presynaptic stimulus was paired with a brief current injection (typically 750 pA for 5 ms; delay of 5 ms between pre- and postsynaptic stimulation)^7^. The second protocol was a high-frequency stimulation/pairing paradigm (HFS-P) that consists of three trains of 100 presynaptic pulses at 100 Hz paired with a postsynaptic depolarization at 0 mV in voltage-clamp (intertrain interval 30 s)^7^,^20^. The third protocol was a ‘spike-timing-dependent plasticity’ protocol (STDP). The pairing involved a presynaptic stimulus and a brief postsynaptic current injection evoking two action potentials, typically around 750 pA for 11 ms. The delivery of the presynaptic stimulation preceded the postsynaptic current injection by 10–11 ms. The pairing was repeated 100 times at 0.166 Hz^20^. In all LTP-induction protocols for single cell experiments we used the same presynaptic stimulation intensity and duration as in test recordings.

### Two-photon imaging

A commercial two-photon laser-scanning Femto2D microscope from Femtonics (Budapest, Hungary) was used. Laser pulses at 810 nm were provided by a Ti:Sapphire femtosecond laser (Cameleon Ultra I, Coherent). For measuring Ca^2+^ signals, green (Fluo 5F) and red (Alexa-Fluor 594) fluorescence were collected during 500 Hz line scans across the spine head and/or its parent dendrite. Fluorescence changes were quantified as the increase in green fluorescence normalized to the average red fluorescence (ΔG/R)^48^. The Ca^2+^ transient peaks were estimated from exponential fits of the fluorescence traces. Fluorescence was collected through the objective (60 × 1.0 NA, Olympus) and the oil immersion condenser (1.4 NA, Olympus) with two pairs of photomultipliers (2 for collecting red band fluorescence and the other 2 for the green band fluorescence). An additional photomultiplier was used to collect the transmitted infrared light. The composition of the intracellular solution for these experiments was in mM: 130 potassium gluconate, 20 HEPES, 2 MgCl_2_, 2 Mg-ATP, 0.3 Na-GTP, 0.25 Fluo-5F and 0.02 Alexa 594. The pH was adjusted to 7.3 and the osmolarity to 290 mOsm. The extracellular solution was the same as in the other experiments and also contained 20 μM bicuculline. Fluorescence data recording started 15 min after attaining the whole-cell configuration.

All data are presented as mean ± standard error of the mean. Statistical tests were performed using Prism 6 (GraphPad Software Inc, La Jolla, Ca, USA) and are described where appropriate in the text.

## Additional Information

**How to cite this article**: Lopez-Rojas, J. *et al*. Plasticity of intrinsic excitability in mature granule cells of the dentate gyrus. *Sci. Rep.* **6**, 21615; doi: 10.1038/srep21615 (2016).

## Supplementary Material

Supplementary Information
