# Supplementary material for: Plasticity of intrinsic excitability in mature granule cells of the dentate gyrus
Source: Sci Rep. 2016 Feb 9;6:21615. doi: 10.1038/srep21615 (PMC4746665; doi:10.1038/srep21615)
Supplement: Supplementary Information [file srep21615-s1.pdf]

Title: Plasticity of intrinsic excitability in mature granule cells of the dentate gyrus

Authors: Jeffrey Lopez-Rojas[a1], Martin Heine[b1] and Michael R. Kreutz[ac1]

[a]Research Group Neuroplasticity, [b]Research Group Molecular Physiology, Leibniz Institute for Neurobiology, Brenneckestrasse 6, D-39118 Magdeburg, Germany, [c]Leibniz Group 'Dendritic Organelles and Synaptic Function', University Medical Center Hamburg-Eppendorf, Center for Molecular Neurobiology, ZMNH, 20251 Hamburg, Germany.

[1]To whom correspondence should be addressed. Email: jeffrey.lopez@lin-magdeburg.de, martin.heine@lin-magdeburg.de or michael.kreutz@lin-magdeburg.de

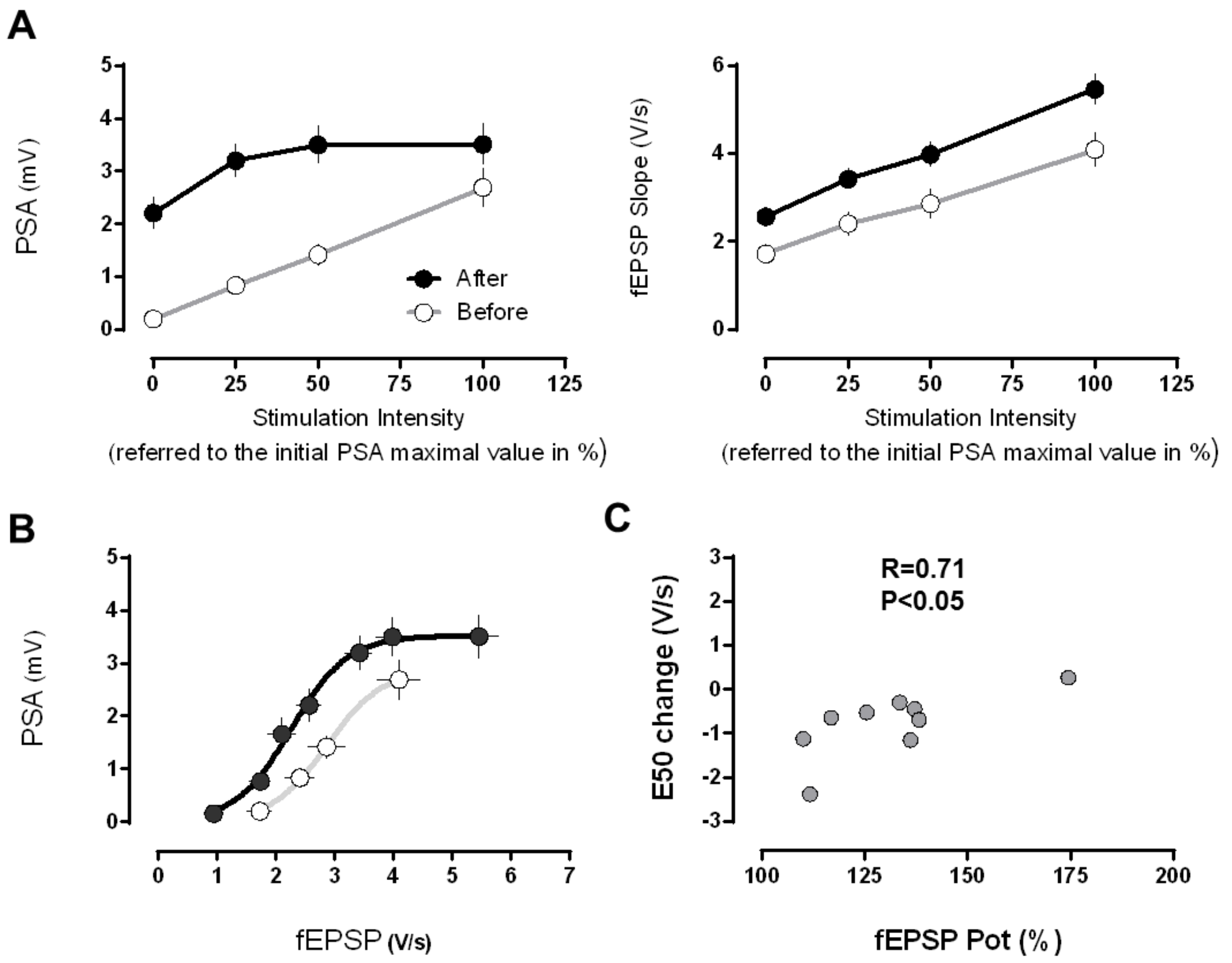

Supplementary Figure S1. Excitability changes are inversely related to synaptic potentiation in the TBS group, as evidenced by input-output curves analysis. **A**, Input-output curves for the population spike amplitude (PSA) and fEPSP-slope were obtained before and 70min after the TBS protocol in 9 out of 13 of the experiments shown in Fig.1 for the TBS group. For every slice four stimulation intensities were used in the “before” curve, corresponding to the maximum PSA, 50% of the maximum, 25% of maximum and PSA threshold (minimal PSA) value. For the “after” curve the field responses to the same stimulation intensities were studied. Additionally 3 new stimulation intensities were used in order to match the 50%, 25% and threshold PSA values previous to TBS. For clarity only the common four stimulation intensities are shown in A. **B**, E-S plot. The PSA values are plotted vs. the corresponding fEPSP values. A significant shift of the E-S curve occurs after TBS protocol. **C**, when plotting the E50 change for every single experiment vs. the fEPSP

potentiation attained, a significant correlation was found ($R=0.71$, $p< 0.05$, Pearson), supporting the analysis in Fig. 1.

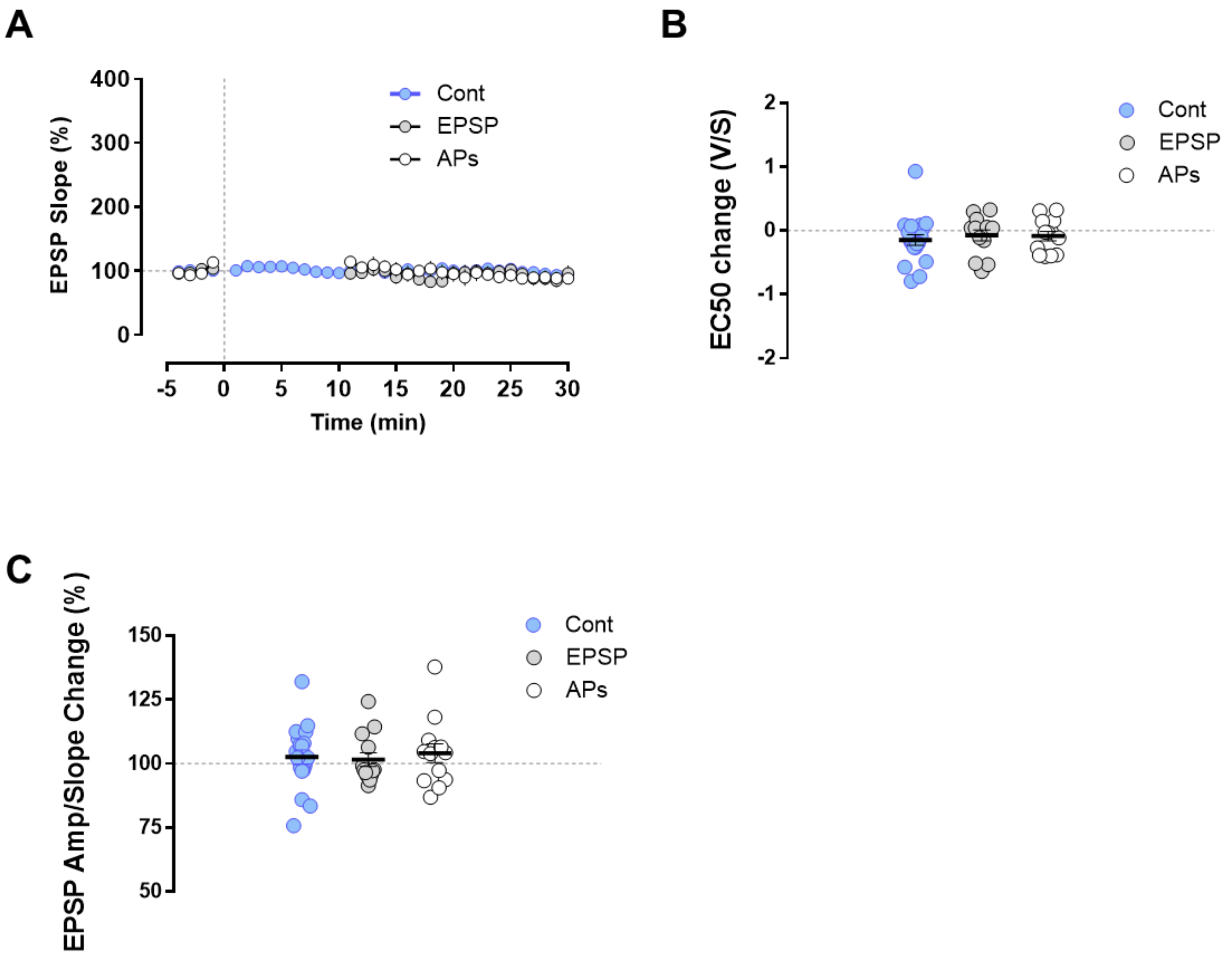

Supplementary Figure S2. . Neither synaptic stimulation (EPSP, n=13) nor postsynaptic action potential firing alone (APs, n=13) at 0.166Hz for 10 min (from time “0” up to 10 min), provoked any statistically significant changes in the time course of the EPSP-slope or in intrinsic excitability. **A,** The time course of EPSP-slope in these two additional control groups was similar to the other control group (Cont, n=20) receiving test stimulation during all the recording period. None of these three control groups showed any significant changes in E50 values **(B)** or EPSP-amplitude/slope relation **(C).** All the recordings were made in presence of 20 µM bicuculline.

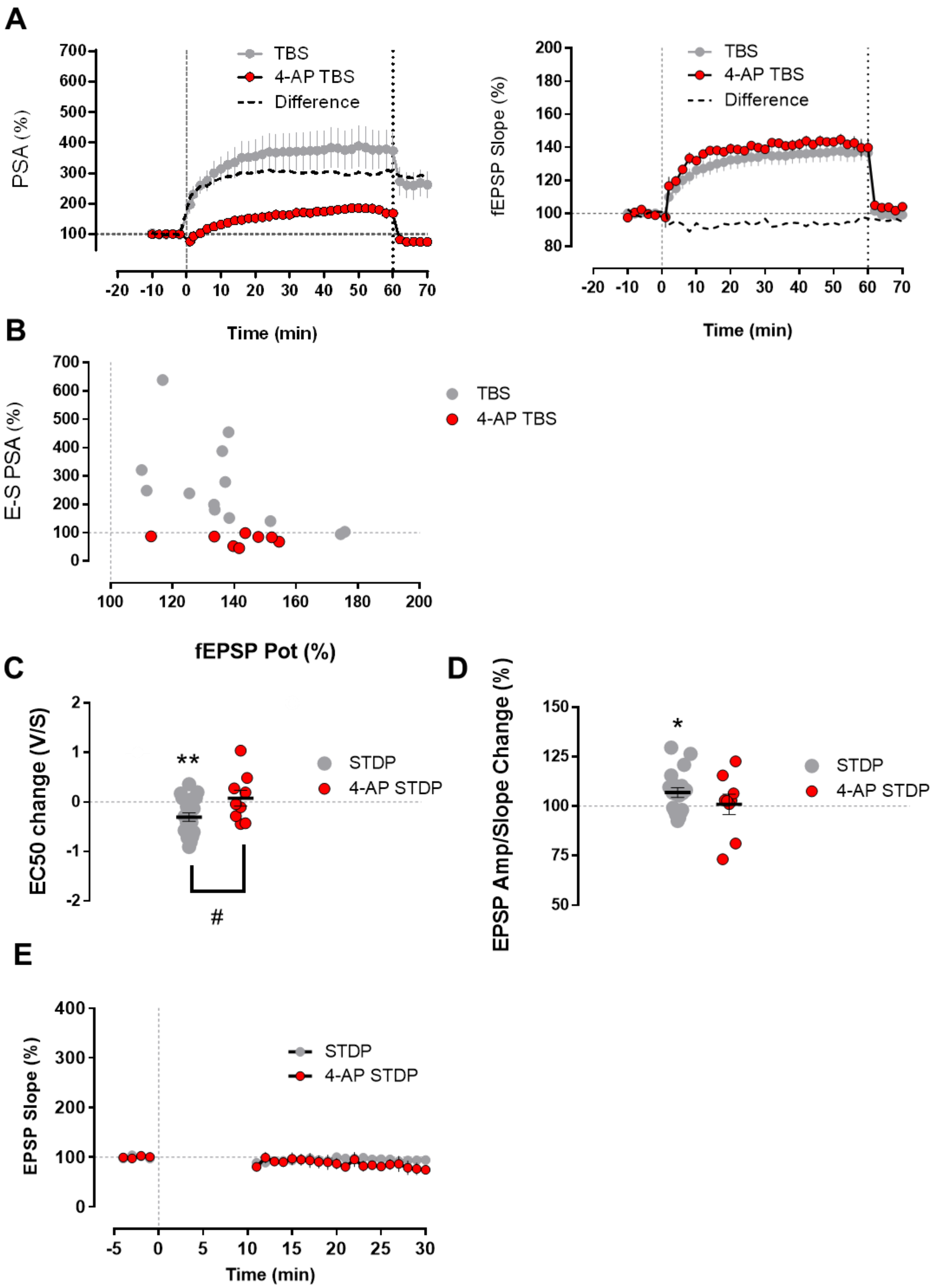
A
PSA (%)
TBS
4-AP TBS
Difference
Time (min)
fEPSP Slope (%)
TBS
4-AP TBS
Difference
Time (min)
B
E-S PSA (%)
TBS
4-AP TBS
fEPSP Pot (%)
C
EC50 change (V/S)
**
#
STDP
4-AP STDP
D
EPSP Amp/Slope Change (%)
*
STDP
4-AP STDP
E
EPSP Slope (%)
STDP
4-AP STDP
Time (min)

Supplementary Figure S3. Excitability changes after TBS and STDP protocols are occluded by the A-type channel blocker 4-aminopyridine. **A**, Time course of population spike amplitude (PSA) and field EPSP-slope. PSA and EPSP values were normalized for every slice to the pre-conditioning baseline. The TBS protocol was applied at time “0” with intact GABAergic inhibition in absence (n=13) or presence of 4-aminopyridine 5mM (n=8, bath applied at least 1h before TBS until the end of the recording period). One hour after TBS protocol, the test stimulation intensity was reduced to match the baseline EPSP-slope values. The remaining PSA potentiation displays the excitability changes not explained by the synaptic potentiation. **B**, Scatter plot of the PSA potentiation that is not explained by the synaptic LTP and the EPSP-slope potentiation at 1 hour after TBS for each experiment. We observed a significant negative correlation between both variables in the TBS group ($R=0.58$, $p< 0.05$, Pearson). This correlation was not present in the 4-AP TBS group. Pharmacological blockade of A-type potassium channels by 4-aminopyridine (bath applied for around 1 hour prior to the STDP protocol and until the end of the recording period) occluded any significant changes in the E-S curve (**C**) or in the EPSP-amplitude/slope relation (**D**) after the STDP protocol. **E**, Time course of the EPSP-slope after STDP protocol in absence (STDP, n=13) or presence of 4-aminopyridine (4-AP STDP, n=9). All the patch clamp recordings were made in presence of 20 µM bicuculline. *,**: $p<0.05$ and $p<0.01$, paired t-test. #: $p<0.05$, t-test.

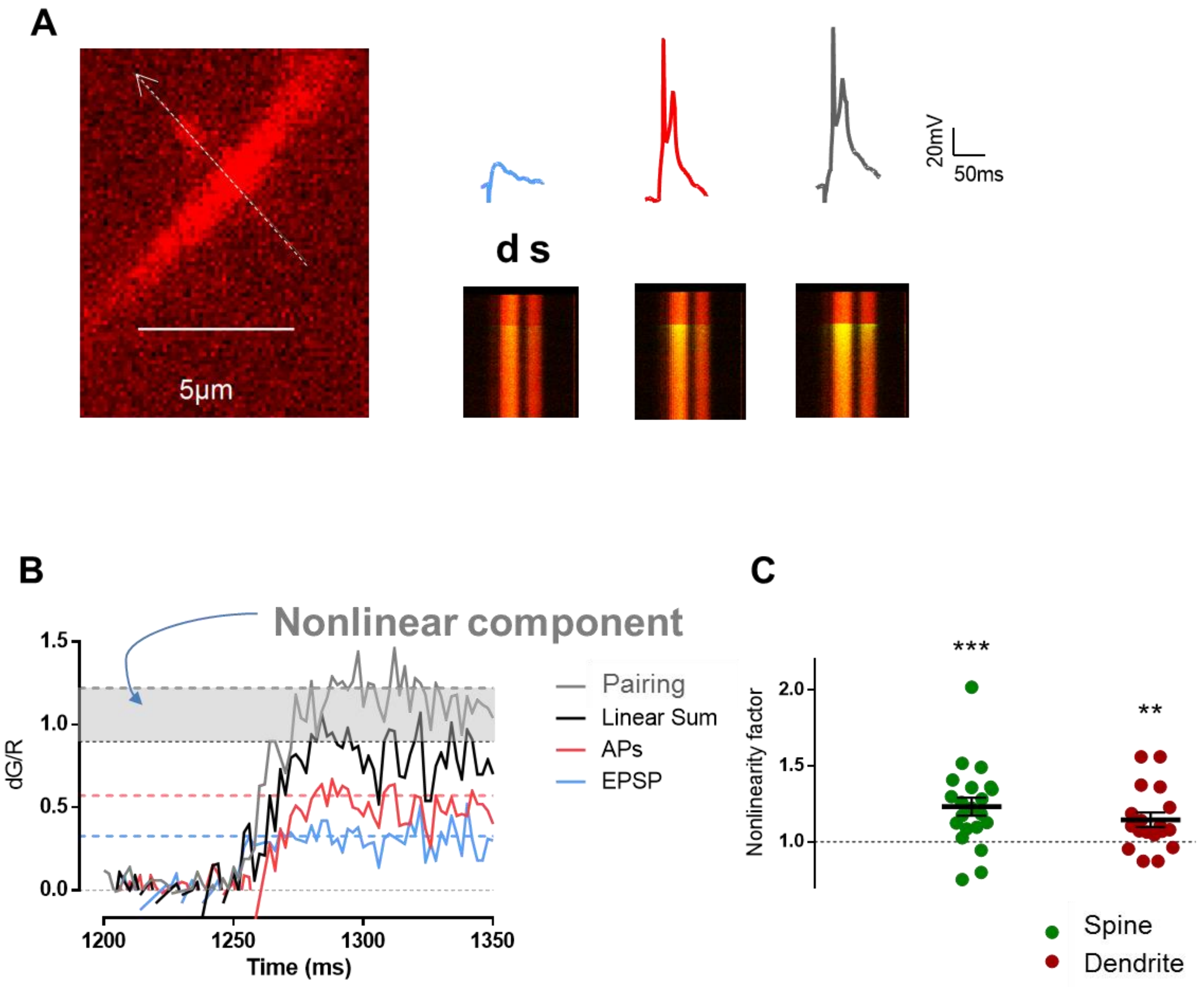

Supplementary Figure S4. Supralinear Ca^{2+} summation in spines and dendrites of mature granule cells during STDP pairing. **A**, Two photon fluorescence image of a spine and shaft dendrite. The position of the line scan through the spine and shaft is indicated by the dashed line. The insets show the somatic voltage recordings and the line scans of the spine (s) and shaft dendrite (d) in response to a sub-threshold EPSP (blue trace), a duplet of action potentials (red) and to a EPSP- action potential duplet pairing (gray). **B**, The nonlinearity factor was calculated as the Ca^{2+} influx during the pairing normalized to the “expected” linear sum of EPSP-evoked and action potential duplet-evoked Ca^{2+} transients. **C**, There was a significant supralinear summation in both spines (n=21) and dendrites (n=18) of mature

granule cells during EPSP-action potential duplet pairings. All recordings were made in the presence of bicuculline. **,***: $p<0.01$ and $p<0.001$ one sample t-test vs. 1.
